# Plugged-in: a Canadian survey of technology ownership, access, use, and attitudes among emergency department patients

**DOI:** 10.3389/fdgth.2025.1507936

**Published:** 2025-06-30

**Authors:** Aisling Curtin Wach, Krutika Joshi, Christina Seo, Pete Wegier

**Affiliations:** ^1^ Humber River Health Research Institute, Humber River Health, Toronto, ON, Canada; ^2^ Department of Family and Community Medicine, University of Toronto, Toronto, ON, Canada; ^3^ Institute of Health Policy, Management and Evaluation, Dalla Lana School of Public Health, University of Toronto, Toronto, ON, Canada

**Keywords:** emergency department, digital technologies, patient survey, digital health, internet

## Abstract

**Introduction:**

Patient-facing digital health technologies have the capacity to remedy some of the challenges faced by overburdened and under-resourced Canadian emergency departments (ED). However, the successful implementation of such innovations is dependent on patients' willingness and ability to access and use digital technologies. Moreover, the potential presence of digital disparities in local communities may create or exacerbate inequitable health outcomes. This study aimed to understand technology ownership, access, use, and attitudes among ED patients of a digitally innovative hospital located in an ethnoculturally diverse, urban area of Toronto.

**Methods:**

An electronic, self-report, cross-sectional survey was conducted in the ED of an urban, community hospital in Toronto. A convenience sample of ED patients over the age of 18 and proficient in English were invited to participate in the survey between January 3rd and February 14th, 2024. Participants responded to a battery of questions and scales (e.g., the Media and Technology Usage and Attitudes Scale; MTUAS) that were adapted as necessary for this study.

**Results:**

The final sample size of 250 participants had a mean age 40.4 ± 16 years, 64.4% were female, and 38% were born outside of Canada. Ownership of at least one digital device was high (97.6%), as was the use of smartphones (96.0%), email (93.6%), text messaging (94.8%), and internet searching (95.6%). Almost all participants had access to the internet (98.0%). Attitudes towards technology were generally positive (mean 4.2/5). There were no significant differences in technology ownership and use based on where participants lived. Few barriers to technology were reported.

**Conclusion:**

Despite concerns of digital disparities in an ethnoculturally diverse area of Toronto, technology ownership, access, and use appear to be pervasive among ED patients, irrespective of where they reside. These findings, coupled with patients' generally positive attitudes towards technology, green-light the exploration of patient-facing digital health tools that utilize the digital technology ED patients already own, access, and use to improve the delivery of emergency care.

## Introduction

Humber River Health (HRH) is North America's first fully digital hospital and one of Canada's largest acute care community hospitals. Situated in the Northwest region of Toronto, Ontario, HRH serves a diverse urban community of over 850,000 people, which encompasses several low-income, high-density neighborhoods that are considered some of the most ethnoculturally diverse in Canada (1). The emergency department (ED) at HRH has not been immune to the challenges reported across Canadian EDs, regarding an increased strain on resources due to a rising demand for healthcare services that is outpacing the capacity of the healthcare system (2). Data collected by the Canadian Institute for Health Information's (CIHI) National Ambulatory Care Reporting System (3) indicates that ED volumes have recovered back to pre-pandemic levels, increasing from 14 million in 2021–2022 to 15.1 million in 2022–2023. Moreover, EDs have observed a change in the acuity (i.e., severity of condition) of patient presentations, such that EDs are receiving a higher proportion of patients with an assigned acuity level requiring urgent or emergent care with rapid intervention and lower proportions of patients with an acuity level requiring less urgent care (3). Thus, EDs are now experiencing a higher volume of patients with complex health needs than prior to the Covid-19 pandemic.

As an innovator in digital healthcare, HRH proposes to explore ways in which patient-facing digital tools may alleviate some of the pressures faced in the ED. Digital health technologies have the capacity to improve the ED experience, particularly for medically stable, low acuity patients seeking urgent care, and support the effective and efficient delivery of healthcare services (4, 5). Moreover, with the rapid progression of technology and generative artificial intelligence (AI), digital health technologies present a cost-efficient solution to alleviating the current pressures and burdens experienced across healthcare systems (6, 7). For example, virtual urgent care programs that use technology patients own or have access to, such as smartphones and computers, can offer potential ED patients a convenient way to receive medical care remotely while simultaneously helping to manage ED patient flow, overcrowding, and hospital resource management. Similarly, the development of AI-enabled virtual triage applications that allow medically stable, non-urgent patients to book optimized timeslots to see ED physicians would also assist in the management of ED patient flow, overcrowding, and resource allocation. However, the successful implementation and adoption of digital technology in a diverse community setting cannot be undertaken without taking digital equity into consideration, as such innovations require patients to be both able and willing to access and use technology (8–14). Moreover, the creation and implementation of patient-facing technology in the ED setting must be concerned with whether it may inadvertently create or perpetuate inequitable health outcomes (10, 15).

Past research indicates that ED populations have high rates of technology ownership and usage. A 2022 Boston-based study published by Goldfine and colleagues (16) found that 93.1% of ED patients used smartphones, in addition to ED patients reporting positive attitudes towards technology (mean 3.9/5). Another study found similarly high mobile device ownership with 91% of ED patients owning a smartphone and 96% owning a cellphone (17). However, a review of the relevant literature indicates that these and other related studies (18, 19) have taken place outside of Canada and as such, their results are representative of ED patients in countries that may have different technology infrastructure, in addition to variations in the availability and structure of programs for increasing accessibility to technology (20). For example, in the United States (US), the Lifeline Assistance program (21) also known as the Obama Phone program, provides low-income individuals and families with free cellphones, voice minutes, text messages, and mobile internet data and has been offering free cellphones to low-income individuals and families for over 15 years. This contrasts with Canada's Connecting Families Benefit (22) which provides low-income individuals and families with free computers and low-cost, high-speed internet. This program only came into effect in November 2023, and participation in this program by low-income people and families is via government invitation only. More generally, the price of Canadian cellphone plans are among the highest in the world, with Canada ranking second place among 51 American, African, Asia Pacific, European, and Middle East countries reviewed by independent telecom research firm, Rewheel (23). In the same review, the median price of smartphone plans in Canada were estimated to be 30% higher than US-based plans.

Looking outside of research specific to technology ownership and use in ED populations, a report (24) exploring an apparent digital divide in Toronto, reported high rates of digital connectivity, with 98% of surveyed Toronto households having internet access and almost all Toronto households having a least one device capable of accessing the internet (smartphone, tablet, computer). However, the report also identified six areas in Toronto with internet access rates lower than 96%, hence, these areas were flagged in the report as being digitally divided. The six areas highlighted are all designated Neighborhood Improvement Areas and one of these areas is encompassed in HRH's immediate catchment area (Humber Summit/Jane and Finch). In addition, anecdotal evidence from ED physicians regarding inequitable technology access, ownership, and usability among our ED population raised concerns about the level of digital equity in our community and the potential negative impact implementing digital health technologies in the ED could have on health outcomes in such a diverse community.

Thus, given the gap in Canadian-based research in the ED setting, in addition to empirical and anecdotal reports of inequitable access to technology in our local community, we undertook a survey of HRH's ED patients to understand their ownership, access, use, and attitudes towards technology.

## Methods

### Study design

An electronic, self-report, cross-sectional survey, deployed in-person via institutionally owned iPads was deemed the most appropriate method of data collection due to the dynamic and complex nature of the ED setting (25). The survey design was guided by the Technology Acceptance Model (TAM) (8), which is a framework for understanding acceptance of technology. Specifically, the TAM posits that an individual's perceived usefulness and perceived ease of use informs their attitudes towards technology, which subsequently influences their behavioral intention to use the technology and their actual technology use. As such, survey questions and response options were selected from relevant validated scales and research literature (Supplementary Material) to assess patients' general access and barriers to technology, technology use, and attitudes. Survey questions were adapted as necessary to both reduce redundancy in participant responding and increase specificity to HRH. ED leadership and key ED clinical staff were consulted on the appropriateness of the study design and survey questions, and face validity was determined via team meetings and survey pilot testing within the larger research team.

### Participants

Patients arriving at HRH's ED who were triaged to waiting areas and private rooms designated for patients with an assigned Canadian Triage and Acuity Score (CTAS) (26) of 3 (requires urgent care), 4 (requires less urgent care), or 5 (requires non-urgent care) were approached by a research staff member about participating in a survey regarding patient barriers to technology, including their technology ownership, usage, and attitudes. These areas were identified by ED clinical staff and management as the most appropriate areas to approach medically stable patients seeking emergent care and contrasts to the Acute and Sub-Acute areas that see patients triaged with a CTAS of 1 (requires resuscitation) or 2 (requires emergent care and rapid medical intervention). Inclusion criteria consisted of being 18 years or older, attending the ED as a patient, and proficiency in English (comfort responding in English was self-determined by patient). Patients presenting with a psychiatric chief complaint, psychological distress, cognitive impairment, previous survey completion, being unable to provide consent (e.g., alcohol or drug impaired), or those who arrived at the ED in police custody were not eligible to participate. In addition, patients receiving acute or sub-acute emergent medical intervention were not approached.

### Data collection

To capture a representative sample of patients from our ED population during the data collection period, recruitment shifts covered the 24 h cycle and seven days of the week. Specifically, recruitment shifts were 8 am–4 pm, 4 pm–12 am, and midnight-8 am, with an entire week dedicated to each shift time. This cycle was repeated until the projected sample size was reached. Posters and brochures explaining the research study were placed throughout the ED to ensure patients understood that research activities were not a part of the care they would receive while attending HRH.

Interested potential participants were screened for study eligibility by a research team member in the O-Zone and Fast-Track waiting areas and private consultation rooms, and those who were deemed eligible to participate were provided an iPad with a preloaded electronic consent form hosted on the Qualtrics survey platform (27). Those who completed the consent form automatically proceeded to the confidential electronic survey via Qualtrics. Upon survey completion, participants were further debriefed about the aim of the study (i.e., to understand patient barriers to technology use). If participants felt some discomfort upon reflecting about their ownership, access, use, and attitudes regarding technology even after they completed the survey, there was a distress center phone number provided on the consent form. All participants who completed the survey received a $5 physical gift card as a token of appreciation. To reduce barriers to participation based on familiarity and comfort with technology, all participants were offered the assistance of the onsite researcher should they have difficulty navigating the electronic survey on their own.

### Measures

#### Demographics

The patient survey assessed a variety of demographic characteristics that have been associated with barriers and attitudes to technology use, such as: age, gender identity, educational level, income, and forward sortation area (FSA; i.e., first three characters of their postal code) (17, 19, 24, 28, 29).

#### Technology ownership, access, and barriers

Participants were asked across two questions to identify what pieces of technology they (1) own and (2) have access to where they live, from a list of commonly owned technologies (smartphone, cellphone, landline, laptop, desktop, tablet, smartwatch). For participants who did not own any digital technologies (as indicated by responding “I do not own any…” or only selecting “landline”), they were asked to indicate why they do not own any from a list of options derived from literature on digital equity, technology acceptance—including perceived usefulness and ease of use, and barriers to technology in older adults (10, 14, 17, 24, 30). Response options covered issues related to affordability, infrastructure, physical and cognitive capacities, self-efficacy, perceived usefulness, ease of use, and privacy/security concerns. Participants were also provided with an open text response option if their reason for not owning technology did not fall into the choices on our predetermined list.

#### Connectivity

Participants' level of connectivity was assessed by asking dichotomous “yes/no” questions for if they had WiFi at home and whether they had a cellular data plan. Participants who answered “no” were then asked to indicate why they did not and were provided with an open text box to register their replies.

#### Technology use and attitudes

Participants' use of technology, in addition to their positive and negative attitudes, were assessed using 31 items from the following seven subscales of the Media and Technology Use and Attitudes Scale (MTUAS (31);: (1) emailing, (2) texting, (3) smartphone, (4) phone calling, (5) internet searching, (6) positive, and (7) negative attitudes scales. Subscales (1)–(5) are usage subscales that use a 10-item frequency subscale (i.e., 1 “never”, 2 “once a month”, 3 “several times a month”, 4 “once a week”, 5 “several times a week”, 6 “once a day”, 7 “several times a day”, 8 “once an hour”, 9 “several times an hour”, 10 “all the time”) to measure how often an individual engages in technology related activities associated with emailing, texting messaging, using a smartphone, phone calling, and internet searching. For example, in the texting subscale, participants are asked to indicate how often they do each of the following activities on their mobile phone: “Send and receive text messages on a mobile phone”, “Check for text messages on a mobile phone”, and “Use your mobile phone during class or work time”.

We slightly modified the MTUAS to include a dichotomous “yes/no” question before each usage subscale, such that participants were asked, “Do you have an email account”, “Do you use text messaging”, “Do you use internet searching”, and so on. This was to remove redundancy in answering questions related to the frequency of behavior a person does not engage in, and thus, reduce the amount of time participants spent answering questions. Participants who answered “no” at the beginning of a subscale were then asked to indicate why they do not engage in this activity from the previously discussed list of barriers, before moving on to the next usage subscale.

Subscales (6) and (7) use a 5-point Likert scale (i.e., 1 “strongly disagree”, 2 “disagree”, 3 “neutral”, 4 “agree”, 5 “strongly agree”) to assess positive and negative attitudes towards media and technology use. Participants were asked to rate their agreement with statements such as, “I feel it is important to be able to access the internet any time I want” (positive attitudes subscale) and “New technology makes people waste too much time” (negative attitudes subscale).

### Data analysis

Only participants whose progress was greater than or equal to 95% were included in the analysis. Demographics such as age, sex, gender, first language, and country of birth were collected and reported as such. For ease of analysis, certain variables were dichotomized such as income, education, relationship status, and living situation. The survey included the income categories (in Canadian dollars): *Less than $30,000*, *$30,000—$60,000*, *$60,000—$100,000*, *$100,000—$150,000*, *$150,000 or more*, and *Don't know/Prefer not to say*. This was split into two groups, with *Less than $30,000* in one category, and the rest of the options in another, with *Don't know/Prefer not to say* excluded in this binarization. This was done to represent low income compared to the rest. For education, the survey categories were: *Some Highschool*, *Highschool*, *Some Post-secondary School*, *College/Bachelor's Degree*, *Graduate Degree*, and *Other*. *Some Highschool*, *Highschool*, and *Other* were grouped to create a split between those with post-secondary education and those without. For relationship status, the survey categories were: *Single, Married/Common Law, Separated/Divorced* and *Widowed*. For some of the analysis, this was split into two groups, with *Married/Common Law* in one group and the rest in the other. The dichotomized categories of living situation were also simplified at times, with *Private rental accommodation with own bedroom* and *Private rental accommodation with shared bedroom* being combined into *Private rental accommodation,* and *Supportive housing*, *Emergency accommodation*, and *Long-term care home* being grouped into an *Other* category due to a small number of participants choosing these categories. This was further binarized to create a split between those living with others and those living alone.

For the MTUAS, numerical values were mapped on to the scales in ascending order as mentioned above and mean scores were calculated by usage type (smartphone usage, text messaging usage, internet searching, emailing usage). Participants who answered “no” to each subscale were recoded as having the lowest usage (a value of 1 “never”) so as not to inflate analyses by accounting for only those participants who use technology. A series of descriptive analyses were used to examine technology access, ownership, and usages. To understand associations between technology usage and attitudes, we used Student's *t*-tests and Pearson correlations.

To visually map participants' area of residence, their reported FSAs were connected to the corresponding FSA codes using the Postal Code Conversion File (32). The FSA codes were then joined with an FSA boundary file to map out areas that participants lived. The number of participants living in each FSA was counted to create a heatmap of locations surrounding HRH. Other EDs nearby were also plotted in relation to HRH to have a better understanding of participant proximity to care. Any participants living outside of the Greater Toronto Area or in an undefined FSA were removed from the geographic analysis. Given the prior report of a digital divide in Toronto (24) and lower rates of connectivity in neighborhoods encompassing HRH's catchment area, a subset of participants living in the Humber Summit/Jane and Finch area (FSAs: M9l, M3N, M3l) were identified to analyze their internet access and technology usage in comparison to the report. All analyses were completed using Python 3.11 (33).

## Results

Between January 3rd and February 13th, 2024, a total of 432 people in the Fast Track and O-zone waiting areas and private examination rooms of HRH's ED were approached about participating in a study about their ownership, usage, and attitudes of technology. Of the 432 people approached, 172 people either declined or were not eligible to participate for the following top three reasons: not interested in participating (*n* = 49), felt too unwell (*n* = 38), not proficient in English (*n* = 37) (see Table 1 for full list). A further 10 participants were excluded as they completed less than 95% of the survey. The final sample consisted of 250 participants (Table 2).

**Table 1:** Participant breakdown of reasons for declining participation or exclusion from the study analysis.

Number of participants (*n* = 182)	Reason for declining/excluding
49	Not interested in participating
38	Felt too unwell
37	Not proficient in English
12	Not patients of the ED
10	Survey not completed
8	Called away by doctor during approach
7	Said maybe later but not reapproached
4	Complained of bad ED experience
4	Not over 18
4	Reason not recorded
3	Being discharged
2	Previously completed survey
1	Privacy concerns
1	Overwhelmed by the ED environment
1	Dislike of technology
1	Communication difficulties

Note: 172 patients either declined or were not eligible to participate at the time of approach. A further 10 were excluded from analysis as they completed less than 95% of the survey.

**Table 2:** Participant demographics.

Variables	Participants (*n* = 250)
Demographic Characteristics	
Age, mean (SD)	40.5 (16)
Female, % (*n*)	64.4 (161)
Post-secondary or higher, % (*n*)	71.6 (179)
$30,000 income or more, % (*n*)^a^	76.9 (143/186)
Single, % (*n*)	50.4 (126)
Living in private rental accommodation, % (*n*)	54.8 (137)
Living with others, % (*n*)	82.8 (207)
First language, % (*n*)	
English	72.4 (181)
Spanish	4.8 (12)
Tagalog	3.2 (8)
Filipino	2.4 (6)
Other^b^	17.2 (43)
Country of birth, % (*n*)	
Canada	38.0 (95)
Philippines	11.2 (28)
India	5.2 (13)
Nigeria	3.6 (9)
Other^c^	42.0 (105)

Note: ^a^ 64 participants reported that they either did not know or did not want to disclose their income and were therefore excluded from this analysis.

^b^ 29 other first languages.

^c^ 51 other countries of birth.

### Demographics

All participants were between 18 and 89 years old, with a mean age of 40.5 ± 16 years (Table 2). We had a majority of female participants (*n* = 161, 64.4%), out of which 5 participants had a gender identity that was not the same as their observed sex at birth. Out of the 89 male participants, 3 had a gender identity different from their observed sex at birth.

Almost half (48.0%) of participants had a college or bachelor's degree as their highest level of education. The majority of participants did not live alone (82.8%) and were either single (50.4%) or married/common law (40.0%). Many participants were born in Canada (38.0%), followed by the Philippines (11.2%) and India (5.2%). This finding highlights the diverse countries of birth of our participants, with 56 different countries represented. The participants overwhelmingly spoke English as their first language (72.4%), and of those that did not, 59 participants spoke English as their second language, bringing the total number of participants that spoke English as their first or second language to 240 (96.0%).

**Figure 1:**
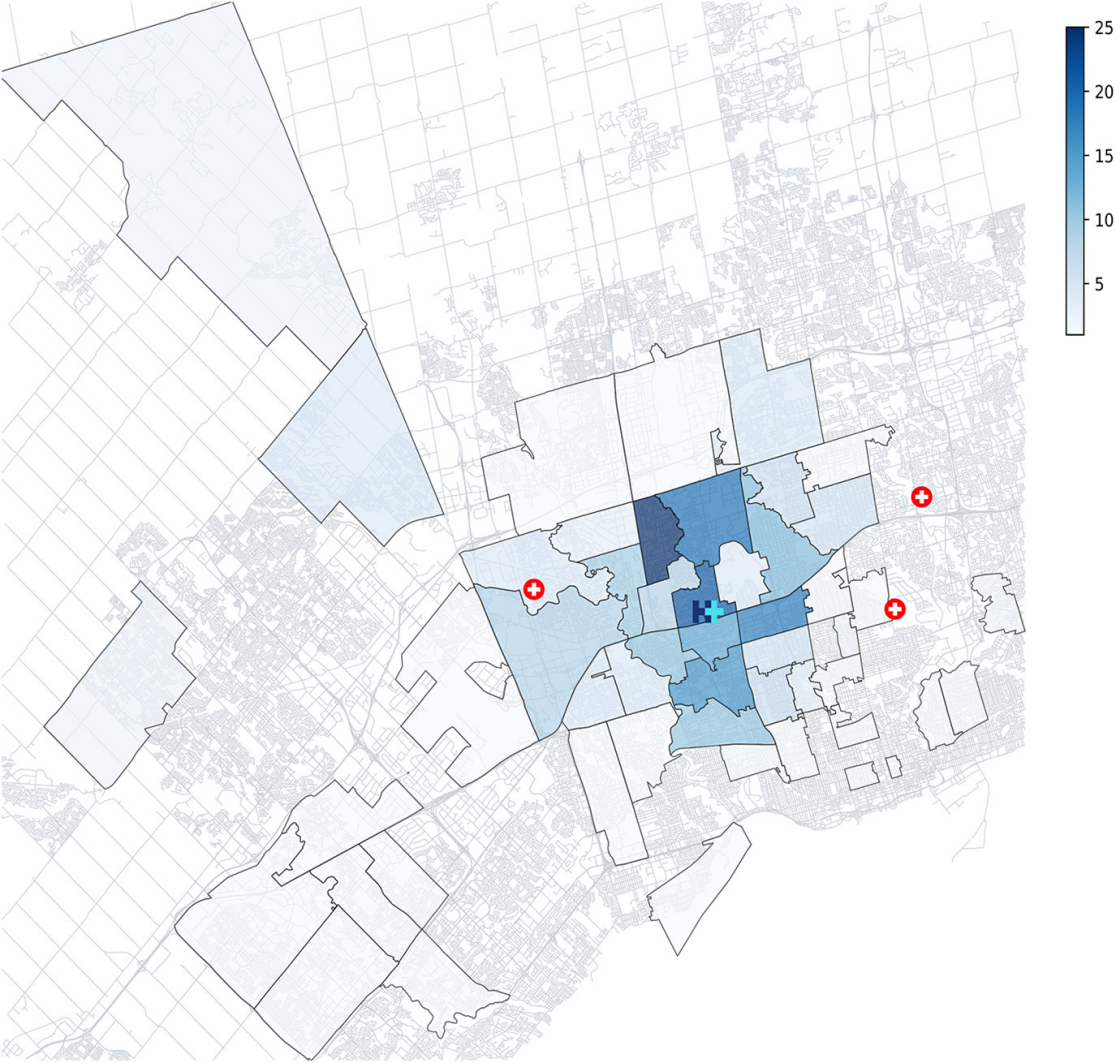
FSA heatmap of participant areas of residence, coloured by number of participants. Most participants lived in areas surrounding HRH, with the most common FSAs being M3N (*n* = 24), M3M (*n* = 20) and M3J (*n* = 18). Other nearby EDs are represented by a red cross, to contextualize where else participants could receive care.

The majority of participants lived in a private rental (54.8%) or a privately owned home (38.8%), and were either driven by a friend or family (39.6%) or drove themselves (24.8%) to the ED. Participants came from many areas of the Greater Toronto Area (and three lived outside of the Greater Toronto Area), but most lived in close proximity to HRH (Figure 1).

### Technology ownership, access, and barriers

Almost all participants (98.0%) indicated that they had internet access (home Wifi or a cellular data plan). The reasons for not having internet access were that it was not useful, it was unavailable in their living situation, it was too expensive, and it was too difficult to navigate. Of the 35 participants that lived in the Humber Summit/Jane and Finch area, 100% of them had internet access.

**Figure 2:**
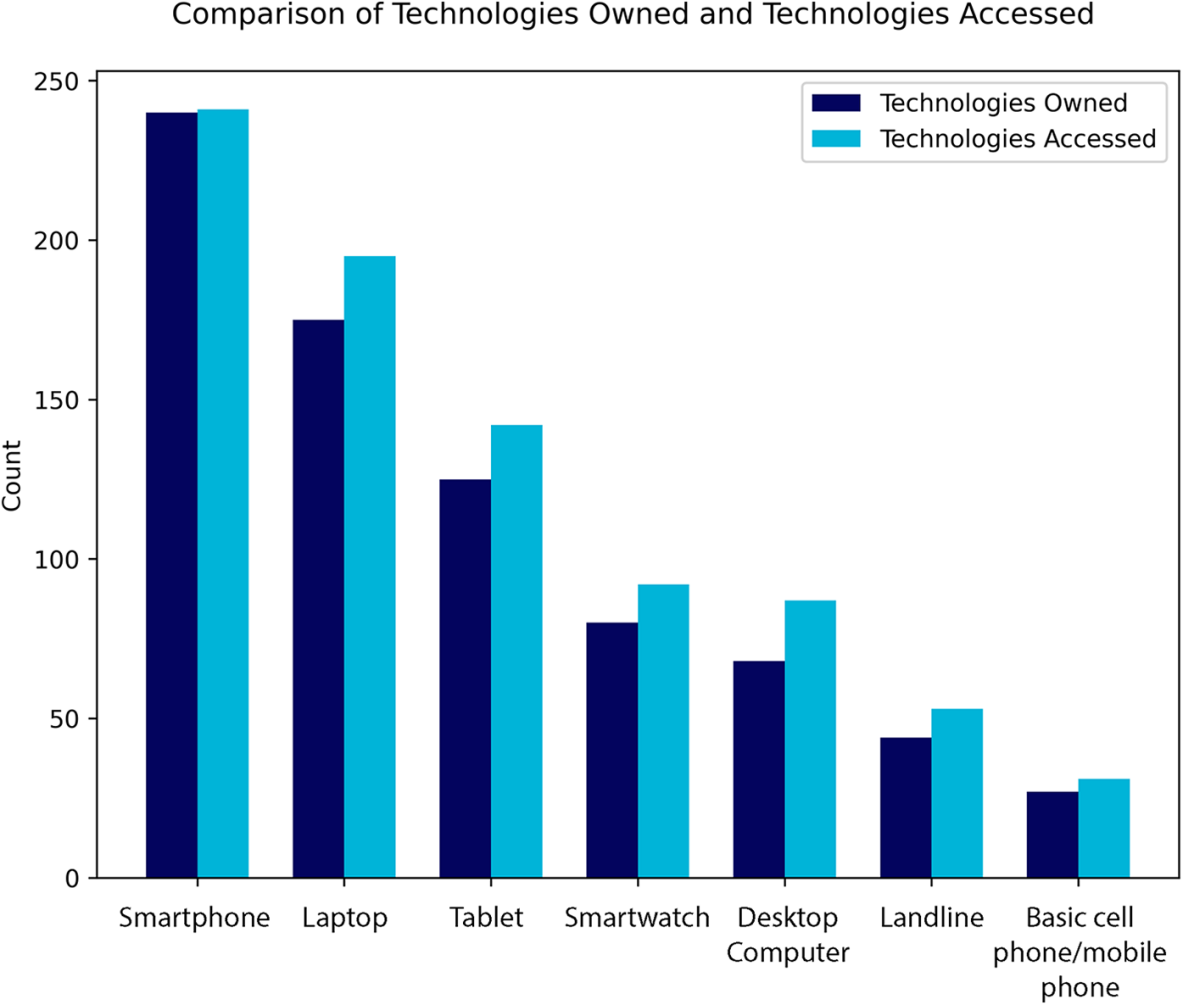
The count of participants owning (dark blue) or having access to (light blue) specific technologies or devices at their place of residence. Digital technologies such as smartphones (n_owned_ = 240, n_accessed_ = 241) and laptops (n_owned_ = 175, n_accessed_ = 195) had higher counts compared to non-digital technologies such as landlines (n_owned_ = 44, n_accessed_ = 53) and basic phones (n_owned_ = 27, n_accessed_ = 31).

97.6% of participants owned at least one digital device (Table 3), which included smartphones, laptops, tablets, and desktop computers (Figure 2). This was 100% when basic cell phones and landlines were included in the devices list.

**Table 3:** Participants' technology ownership, access, and usage.

Variables	Participants (*n* = 250)
Technology ownership and access	
Ownership of technology, % (*n*)	97.6 (244)
Access to technology, % (*n*)	97.2 (243)
Wi-fi in home, % (*n*)	96.4 (241)
Cellular data plan, % (*n*)	96.4 (241)
Technology usage	
Smartphone, % (*n*) (mean usage score^a^)	96.0 (240) (6.7)
Email, % (*n*) (mean usage score)	93.6 (234) (6.5)
Text, % (*n*) (mean usage score)	94.8 (237) (7.5)
Internet search, % (*n*) (mean usage score)	95.6 (239) (6.6)

Note: ^a^ Mean usage scores are calculated out of a 10-point usage scale.

### Technology use and attitudes

96% of participants used smartphones (Table 3). We found a significant relationship (*r* = −0.57, *p* < 0.001) between age and smartphone usage, with older age associated with lower smartphone usage. There was a similar trend between age and text messaging use (*r* = −0.56, *p* < 0.001), age and internet searching (*r* = −0.46, *p* < 0.001), and age and emailing (*r* = −0.34, *p* < 0.001). All of these usages also significantly increased when a participant had an income that was greater than $30,000 (emailing use *t*(184) = −4.80, *p* *<* 0.001; text messaging use *t*(184) = −5.61, *p* *<* 0.001; phone calling use *t*(184) = −2.68, *p* *=* 0.008; smartphone use *t*(183) = −3.92, *p* *<* 0.001; internet searching use *t*(184) = −2.28, *p* *=* 0.024) and had some amount of post-secondary education or higher (emailing use *t*(248) = −4.58, *p* *<* 0.001; text messaging use *t*(248) = −3.44, *p* *<* 0.001; phone calling use *t*(248) = −2.74, *p* *=* 0.007; smartphone use *t*(246) = −3.00, *p* *=* 0.003; internet searching use *t*(248) = −2.93, *p* *=* 0.004). Across all sub-scales, the main reason for non-use was that it was not useful to them. However, other common reasons included privacy/security concerns, difficulty with navigation, and finding its use too time consuming. On the 10-point usage scales, the mean scores were 6.8 for smartphone usage, 7.5 for text messaging usage, 6.6 for internet searching, and 6.5 for emailing usage (Table 3).

**Figure 3:**
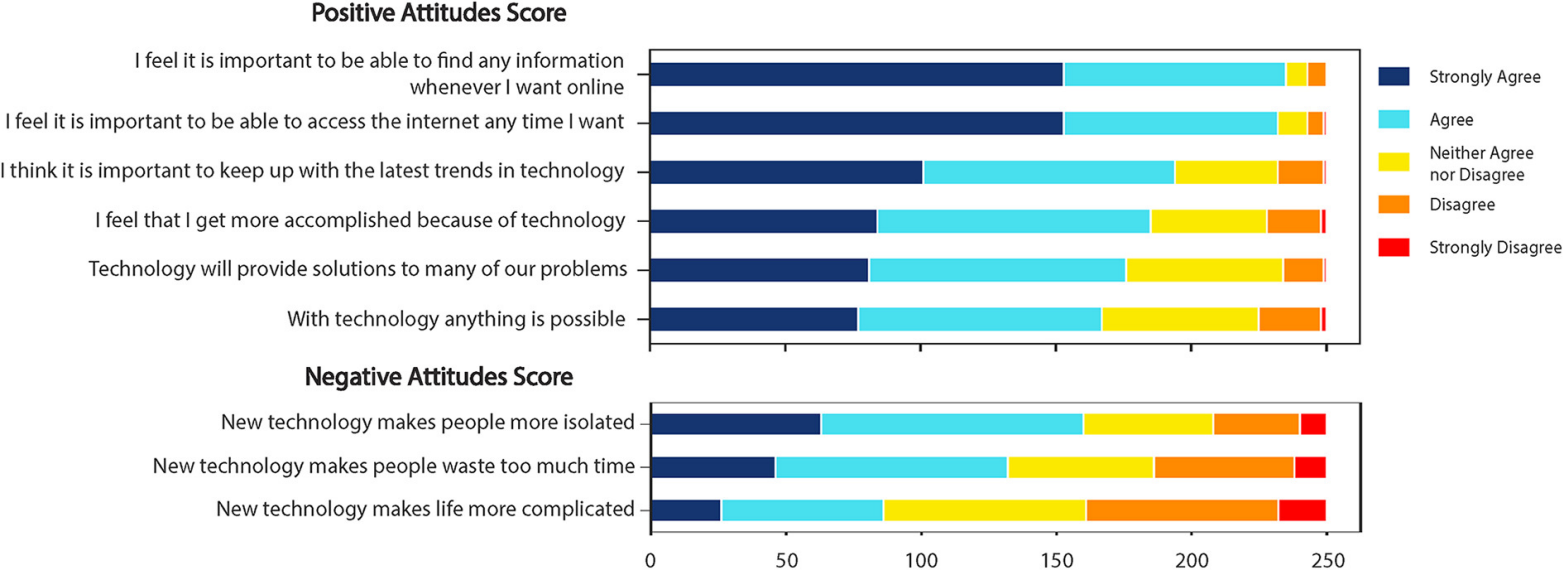
Positive and negative attitudes towards technology participant scores, using terms from the Media technology usage and attitudes scale (MTUAS). Most participants have a positive outlook on technology, with more than 60% (>150/250) agreeing or strongly agreeing to the questions in the positive attitudes subscale.

Overall, most participants have a positive outlook on technology, with more than half agreeing or strongly agreeing to questions about the importance of technology in their lives, the ability to access technology at any given time, and the possibility and accomplishment brought about by technology. Participants also agreed with some of the more negative views of technology; more than half agreed that technology can make people waste time and feel more isolated (Figure 3). We also saw that positive attitudes to technology decreased as participants got older (*r* = −0.25, *p* < 0.001), increased with post-secondary education or higher [*t*(248) = −2.61, *p* = 0.01], had no correlation to income, increased with frequency of technology use (Smartphone usage: *r* = 0.47, *p* < 0.001; Emailing usage: *r* = 0.46, *p* < 0.001; Text messaging usage: *r* = 0.39, *p* < 0.001; Internet searching usage: *r* = 0.47, *p* < 0.001), and decreased for those without access to home [*t*(248) = −3.1, *p* = 0.002] or mobile internet [*t*(248) = −3.6, *p* < 0.001].

## Discussion

The rapid advancement of digital technologies and generative AI presents a promising solution to remedying some of the current challenges faced across healthcare systems, particularly, in the ED setting. Moreover, the high level of technology ownership and use found in prior studies of ED patients suggests that those who seek to develop digital health tools should consider the inclusion of patient-facing digital solutions. However, caution should also be exercised to ensure the implementation of such patient-facing digital health technologies do not create or further deepen health inequities as a consequence of digital disparities within local communities. Our investigation elucidated that technology ownership and access is pervasive among patients attending a Canadian ED, located in an ethnoculturally diverse region of Toronto, in addition to technology usage being high. Furthermore, patients indicated many positive attitudes towards the usefulness of technology which align with the implementation and adoption of digital health tools, including its capacity to provide solutions to many problems, the possibilities technology presents, and accomplishing more due to technology.

Our data on technology usage and attitudes are in line with Goldfine et al.'s (16) study that explored ED patients' technology usage in two academic, urban, medical centers in Boston. In their study, patients were surveyed on their smartphone, text messaging, and email usage by also using the MTUAS. Our participants had similar but slightly higher mean usage scores—6.7/10 vs. 6.4/10 for smartphone use, 7.5/10 vs. 7.2/10 for text messaging use, and 6.5/10 vs. 5.9/10 for emailing use. Comparing the two patient cohorts, we had a similar proportion of females surveyed (64.4% vs. 61.8%) and a slightly younger population (mean age 40.4 vs. 47.2). Given our findings of a significant relationship between age and technology usage, it is unsurprising that our mean scores are slightly higher than what Goldfine and colleagues found. These similar results further support our findings that our patients are not technologically disadvantaged compared to other groups. Patient attitudes towards technology were also similar across the two studies, with our study's mean scores being slightly higher for both positive (4.2/5 vs. 3.9/5) and negative attitudes (3.4/5 vs. 3.2/5).

Notably, our results indicated that technology ownership, access, and use is high regardless of where in Toronto patients reside. As previously mentioned, a report by Andrey et al. (24) highlighted areas of Toronto with lower rates of internet access (<96%), one of these being the Humber Summit/Jane and Finch area which comprises three FSAs near HRH. Our results did not reflect this divide, as participants living in those areas had a higher rate of internet access than our total group (100% vs. 98%). Thus, given the high level of access found in our sample, our data does not corroborate Andrey and colleagues' findings. After our analysis was completed, an updated report was released (34), which removed the Jane and Finch area from the list of low internet access areas. Although this aligns closer with our findings, we still found higher rates of internet access in the Humber Summit area compared to this report. In addition, our study found a perceived lack of usefulness to be the primary reason for why participants did not have internet access, irrespective of where they lived. This finding also deviates from Lockhart & Andrey's (34) report which found cost to be the biggest barrier to internet access among respondents. Thus, concerns about a digital divide in internet access does not appear to be relevant to our ED patient population, nor do we foresee it being a barrier to the implementation of patient-facing digital tools requiring internet access.

Given our findings, it is important to note some of the limitations of this current investigation. First, it is possible that patients who are not interested in technology or who are uncomfortable using it, such as older adults or those with low digital confidence, were more likely to decline study participation (35). Paper-based surveys were not offered as they posed an inherent risk in the busy and transient environment of the ED as they are easily lost, misplaced, or inadvertently viewed by others, which raises concerns about confidentiality. In contrast, iPads allowed participants to enter responses directly or with assistance into a secure, encrypted digital platform with a reduced risk of data being physically visible if the iPad was left unattended or the patient moved around. Protective cases on the iPads offered shielding from others nearby; the screen could be dimmed, and the iPad screen locked after 60 s if left unattended. It is also possible that participants responded in socially desirable ways and over reported their positive attitudes and use to appear technologically savvier. In turn, these biases may account for the low reporting of barriers to technology use. However, steps were taken in the administration of the survey to mitigate against such effects For example, during the initial approach, the researcher always offered to complete the survey on the iPad with the patient and to normalize differences in technology use, it was explained in plain language to all approached patients that all perspectives on technology are important including those who do not readily use it. In addition, survey response choices offered non-usage options (e.g., “Never”) and survey questions were worded in a value neutral way. It should also be noted that only one patient declined participation by explicating they dislike technology. Nonetheless, self-selection bias and socially desirable responding remains a challenge and limitation in research using self-report methods. Future researchers should carefully weigh the risks and benefits of electronic vs. paper-based surveys considering the environmental context, participant mobility, privacy concerns, and technological comfort, and offer both options when feasible to further reduce barriers to participation.

Second, a requirement for study eligibility was proficiency in English. Therefore, our results may not be reflective of all patients in our community who do not speak English well. However, a firm strength of this investigation is the diversity of participants sampled, with 56 different countries of birth reported. Thus, the requirement for proficiency in English does not necessarily preclude the representation of diversity within our sample population. Future research should consider the inclusion of measuring length of time in Canada, generational status (e.g., first generation, second generation Canadian), and cultural background to more fully identify ethnocultural representation within their samples that country of birth alone is unable to comprehensively capture.

Third, as the primary goal of this study was to understand technology access, use, and attitudes in our specific ethnoculturally diverse ED population in Northwestern Toronto, our findings may not be generalizable to other patient populations or geographical regions. Although our findings are in agreement with past research exploring technology access, use, and attitudes in ED patients, future research is required to further contextualize technology access, use and attitudes, particularly, in rural and suburban populations.

Fourth, the validated scales used for the purposes of this survey do not capture newer usage of technologies such as voice notes (which may be used in replacement of text messaging or phone calls) and generative AI. The inclusion of such uses may provide an insight to the number of *early adopters* in our patient population and therefore, a more nuanced understanding of patients' willingness and ability to use not only everyday technologies, but new and innovative ones also.

Fifth, this survey measured attitudes towards technology in general, but it is possible that patients hold different attitudes when asked to consider a particular piece of technology or health related technologies, specifically. Furthermore, our study focused on technology ownership and use alone which may not fully capture patients' capacity to engage with digital health tools. As our study was intentionally designed to be a first step to understanding the feasibility of digital health interventions in our patient population, we only assessed barriers to access and use among those who reported not having access. Therefore, future research should be targeted at understanding patients' attitudes towards specific digital health technologies prior to implementation and build on this foundation to explore quality of access and barriers to using specific tools in greater depth, including issues related to infrastructure, affordability, and digital literacy among those who own and have access to technology. By exploring these challenges in further depth via focus groups and extended surveying, established theoretical frameworks such as the TAM can be applied for more advanced analysis of the factors influencing technology adoption. This in turn may support the co-design, testing, and implementation of patient-facing digital health technologies and the tailoring of interventions to address the needs of diverse and underrepresented populations.

## Conclusion

Despite concerns of digital disparities in an ethnoculturally diverse area of Toronto, our study found that technology ownership, access, and use is pervasive among ED patients, irrespective of where they reside. Moreover, ED patients' attitudes towards technology are generally positive. Thus, this study bolsters past findings of ubiquitous technology ownership and positive attitudes among ED patients and underscores the fertile ground for exploring patient-facing digital health tools that utilize the digital technology ED patients already own, access, and use to improve the effective and efficient delivery of emergency care. Given that this study was born out of a concern for digital equity within our own patient population, we encourage future researchers to remain vigilant to barriers to digital access within their own communities, with the aim to further inform equitable digital health interventions.

## Data Availability

The raw data supporting the conclusions of this article will be made available by the authors, without undue reservation.
