# Supplementary material for: Plugged-in: a Canadian survey of technology ownership, access, use, and attitudes among emergency department patients
Source: Front Digit Health. 2025 Jun 30;7:1507936. doi: 10.3389/fdgth.2025.1507936 (PMC12256449; doi:10.3389/fdgth.2025.1507936)
Supplement: Supplementary file 1 [file Datasheet1.pdf]

**Supplementary Material**

Please tell us a bit about yourself.

1. **How old are you?**

2. **What is your sex at birth?**
   - ❑ Female
   - ❑ Male

3. **What gender do you identify as?**
   - ❑ Man
   - ❑ Woman
   - ❑ Non-binary/third gender
   - ❑ Prefer not to say

4. **What is your current relationship status?**
   - ❑ Single
   - ❑ Married/common law
   - ❑ Separated/ divorced
   - ❑ Widowed

5. **Which of the following best describes your current living situation?**
   - ❑ Alone
   - ❑ With others
   - ❑ Other (Please specify): __________

6. **Which of the following best describes where you live?**
   - ❑ Private rental accommodation with own bedroom
   - ❑ Private rental accommodation with shared bedroom
   - ❑ Privately owned home (e.g., you or a member of your household own the home)
   - ❑ University/college campus accommodation
   - ❑ Community housing
   - ❑ Supportive housing

- ❑ Assisted living accommodation
- ❑ Long-term care home
- ❑ Emergency accommodation (e.g., shelter)
- ❑ Other (please specify): __________

7. **What are the first three characters of your postal code (e.g., M3M?**

8. **What is your primary language?**

9. **Are you fluent in a second language?**
   - ❑ Yes
   - ❑ No

**6A. If yes, what language? [Display if yes above]**

10. **What is your current total household income?**
    - ❑ Less than $30,000
    - ❑ $30,000 - $60,000
    - ❑ $60,000 - $100,000
    - ❑ $100,000 - $125,000
    - ❑ $150,000 or more
    - ❑ Don't know / Prefer to not say

11. **What is the highest level of education you have completed?**
    - ❑ Some high school
    - ❑ Highschool
    - ❑ Some post-secondary
    - ❑ College / Bachelor's Degree
    - ❑ Graduate Degree
    - ❑ Other (Please specify): __________

12. **In what country were you born?**

13. **How did you arrive to the ED?**

- ❑ Drove yourself
- ❑ Family/friend drove you
- ❑ Taxi (including ride share apps like Uber, Lyft)
- ❑ Public transport (e.g., bus, subway)
- ❑ Ambulance
- ❑ Police escort
- ❑ Walked
- ❑ Other (Please specify): __________

The next few questions ask about your ownership and access to different pieces of technology.

14. **Ownership of technologies:**

Please check off all the pieces of technology that **you own**

- ❑ Smartphone (e.g., android, iPhone)
- ❑ Basic cellphone/mobile phone (i.e., a mobile phone without apps and no/limited internet access)
- ❑ Landline
- ❑ Laptop
- ❑ Tablet (e.g., iPad, Samsung Galaxy tab, Amazon Fire Tablet)
- ❑ Desktop Computer
- ❑ Smartwatch (i.e., mobile device worn on wrist)
- ❑ I do not own any of the above

**14a. Which of the following best describes why you do not own any digital technologies? [Display for those who choose landline, or I do not own any of the above] [Responses will appear in random order]**

- ❑ Too expensive
- ❑ Home internet is too slow in the area I live to own a digital device
- ❑ Too difficult to navigate/use
- ❑ I don't know how to use them
- ❑ I find it difficult to perform subtle hand movements such as clicking a mouse and typing
- ❑ I have hearing issues
- ❑ I have vision issues (e.g., eye irritation, blurred vision, near sighted etc.)
- ❑ I have cognitive challenges (e.g., memory issues, difficulty concentrating)
- ❑ I am concerned about my privacy and security
- ❑ Not useful in my daily life/ I do not need it
- ❑ Other (please specify): __________

15. **Access to technologies:**

Please check off all the pieces of technology that you have **access** to where you live

- ❑ Smartphone (e.g., Android, iPhone)
- ❑ Basic cellphone/mobile phone (i.e., a mobile phone without apps and no/limited internet access)
- ❑ Landline
- ❑ Laptop
- ❑ Tablet (e.g., iPad, Samsung Galaxy tab, Amazon Fire Tablet)
- ❑ Desktop Computer
- ❑ Smartwatch (i.e., mobile device worn on wrist)
- ❑ I do not own any of the above

16. **Do you have WIFI internet at home (i.e., home internet)?**
    - ❑ Yes
    - ❑ No

**16a. If no, why not? [Display if answer no]**

17. **Do you have cellular data on your smartphone/ cell phone? [do not display if participant does not own digital technologies – these participants skip to Attitudes subscale]**
    - ❑ Yes
    - ❑ No

**17a. If no, why not [Display if answer no]**

18. **Media and Technology Use and Attitudes Scale (adapted from Rosen et al., 2013 by adding in yes/no qs before each section. 7 out of 15 subscales used)**

**Usage. subscales**

**10-point frequency scale (with scoring in parentheses):**

Never (1), Once a month (2), Several times a month (3), Once a week (4), Several times a week (5), Once a day (6), Several times a day (7), Once an hour (8), Several times an hour (9), All the time (10)

**Do you have an email account:**

- ❑ Yes
- ❑ No **[Display 18a if answer no, skip 18a if yes]**

**18a. Which of the following best describes why you do not have an email account? [Responses will appear in random order]**

- ❑ Too expensive
- ❑ My home internet is too slow to send emails
- ❑ Too difficult to navigate/use
- ❑ I don't know how to use it
- ❑ I find it difficult to perform subtle hand movements such as clicking a mouse and typing
- ❑ I have hearing issues
- ❑ I have vision issues (e.g., eye irritation, blurred vision, near sighted etc.)
- ❑ I have cognitive challenges (e.g., memory issues, difficulty concentrating)
- ❑ I am concerned about my privacy and security
- ❑ Not useful in my daily life/ I do not need it
- ❑ Other (please specify): __________

**Please indicate how often you do each of the following e-mail activities on any device (mobile phone, laptop, desktop, etc.)**

1. E-mailing subscale) Send, receive and read e-mails (not including spam or junk mail).
2. (E-mailing subscale) Check your personal e-mail.
3. (E-mailing subscale) Check your work or school e-mail.
4. E-mailing subscale) Send or receive files via e-mail.

**Do you use text (including Whatsapp text messaging, Apple iMessage):**

- ❑ Yes
- ❑ No **[Display 18b if no; skip 18b if yes]**

**18b. Which of the following best describes why you do not use text (including Whatsapp text messaging, Apple iMessage) [Responses will appear in random order]**

- ❑ Too expensive
- ❑ I have poor phone reception where I live
- ❑ Too difficult to navigate/use
- ❑ I don't know how to use it
- ❑ I find it difficult to perform subtle hand movements such as clicking a mouse and typing
- ❑ I have hearing issues
- ❑ I have vision issues (e.g., eye irritation, blurred vision, near sighted etc.)
- ❑ I have cognitive challenges (e.g., memory issues, difficulty concentrating)
- ❑ I am concerned about my privacy and security
- ❑ Not useful in my daily life/ I do not need it
- ❑ Other (please specify): __________

**Please indicate how often you do each of the following activities on your mobile phone. [only those who choose yes, answer]**

5. (Text messaging subscale) Send and receive text messages on a mobile phone.
6. (Text messaging subscale) Check for text messages on a mobile phone.
7. (Text messaging subscale) Use your mobile phone during class or work time.

**Do you use phone calling:**

- ❑ Yes
- ❑ No **[Display 18c if no; skip 18c if yes]**

**18c. Which of the following best describes why you do not use phone calling? [Responses will appear in random order]**

- ❑ Too expensive
- ❑ I have poor phone reception where I live
- ❑ Too difficult to navigate/use
- ❑ I don't know how to use it

- ❑ I find it difficult to perform subtle hand movements such as clicking a mouse and typing
- ❑ I have hearing issues
- ❑ I have vision issues (e.g., eye irritation, blurred vision, near sighted etc.)
- ❑ I have cognitive challenges (e.g., memory issues, difficulty concentrating)
- ❑ I am concerned about my privacy and security
- ❑ Not useful in my daily life/ I do not need it
- ❑ Other (please specify): __________

**Please indicate how often you do each of the following activities on your mobile phone. [only those who choose yes, answer]**

8. (Phone calling subscale) Make and receive mobile phone calls.
9. (Phone calling subscale) Check for voice calls on a mobile phone.

**Do you use a smartphone**

- ❑ Yes
- ❑ No **[Display 18d if no; skip 18d if yes)**

**18d. Which of the following best describes why you do not use a smartphone? [Responses will appear in random order]**

- ❑ Too expensive
- ❑ Home internet is too slow in my area to own a smartphone
- ❑ Too difficult to navigate/use
- ❑ I don't know how to use them
- ❑ I find it difficult to perform subtle hand movements such as clicking a mouse and typing
- ❑ I have hearing issues
- ❑ I have vision issues (e.g., eye irritation, blurred vision, near sighted etc.)
- ❑ I have cognitive challenges (e.g., memory issues, difficulty concentrating)
- ❑ I am concerned about my privacy and security
- ❑ Not useful in my daily life/ I do not need it
- ❑ Other (please specify): __________

**Please indicate how often you do each of the following activities on your smartphone. [only those who choose yes, answer]**

10. (Smartphone usage subscale) Read e-mail on a mobile phone.
11. (Smartphone usage subscale) Get directions or use GPS on a mobile phone.
12. (Smartphone usage subscale) Browse the web on a mobile phone.
13. (Smartphone usage subscale) Listen to music on a mobile phone.
14. (Smartphone usage subscale) Take pictures using a mobile phone.
15. (Smartphone usage subscale) Check the news on a mobile phone.
16. (Smartphone usage subscale) Record video on a mobile phone.
17. (Smartphone usage subscale) Use apps (for any purpose) on a mobile phone.
18. (Smartphone usage subscale) Search for information with a mobile phone.

**Do you use internet searching?**

- ❑ Yes
- ❑ No **[Display 18e if no; skip 18e if yes]**

**18e. Which of the following best describes why you do not use internet searching? [Responses will appear in random order]**

- ❑ Too expensive
- ❑ Home internet is too slow in my area for internet searching
- ❑ Too difficult to navigate/use
- ❑ I don’t know how to use it
- ❑ I find it difficult to perform subtle hand movements such as clicking a mouse and typing
- ❑ I have hearing issues
- ❑ I have vision issues (e.g., eye irritation, blurred vision, near sighted etc.)
- ❑ I have cognitive challenges (e.g., memory issues, difficulty concentrating)
- ❑ I am concerned about my privacy and security
- ❑ Not useful in my daily life/ I do not need it
- ❑ Other (please specify): __________

**Please indicate how often do you do each of the following activities? [only those who choose yes, answer]**

19. (Internet searching subscale) Search the Internet for news on any device.
20. (Internet searching subscale) Search the Internet for information on any device.
21. (Internet Searching Subscale) Search the Internet for videos on any device.
22. (Internet searching subscale) Search the Internet for images or photos on any device.

**Attitudes. subscales**

**5-point Likert scale for all items (with scoring in parentheses)**

Strongly agree (5), Agree (4), Neither agree nor disagree (3), Disagree (2), Strongly disagree (1)

**Please rate your agreement with the following statements:**

23. (Positive attitudes) I feel it is important to be able to find any information whenever I want online.
24. (Positive attitudes) I feel it is important to be able to access the Internet any time I want.
25. (Positive attitudes) I think it is important to keep up with the latest trends in technology.
26. (Positive attitudes) Technology will provide solutions to many of our problems.
27. (Positive attitudes) With technology anything is possible.
28. (Positive attitudes) I feel that I get more accomplished because of technology.
29. (Negative attitudes) New technology makes people waste too much time.
30. (Negative attitudes) New technology makes life more complicated.
31. (Negative attitudes) New technology makes people more isolated.
